# Validity of Two Consumer Multisport Activity Tracker and One Accelerometer against Polysomnography for Measuring Sleep Parameters and Vital Data in a Laboratory Setting in Sleep Patients

**DOI:** 10.3390/s22239540

**Published:** 2022-12-06

**Authors:** Mario Budig, Riccardo Stoohs, Michael Keiner

**Affiliations:** 1 Department of Sports Science, German University of Health & Sport, 85737 Ismaning, Germany; 2 Somnolab, 44263 Dortmund, Germany

**Keywords:** wearables, actigraphy, polysomnography, validity, sleep, self-tracking, mHealth

## Abstract

Two commercial multisport activity trackers (Garmin Forerunner 945 and Polar Ignite) and the accelerometer ActiGraph GT9X were evaluated in measuring vital data, sleep stages and sleep/wake patterns against polysomnography (PSG). Forty-nine adult patients with suspected sleep disorders (30 males/19 females) completed a one-night PSG sleep examination followed by a multiple sleep latency test (MSLT). Sleep parameters, time in bed (TIB), total sleep time (TST), wake after sleep onset (WASO), sleep onset latency (SOL), awake time (WASO + SOL), sleep stages (light, deep, REM sleep) and the number of sleep cycles were compared. Both commercial trackers showed high accuracy in measuring vital data (HR, HRV, SpO_2_, respiratory rate), r > 0.92. For TIB and TST, all three trackers showed medium to high correlation, r > 0.42. Garmin had significant overestimation of TST, with MAE of 84.63 min and MAPE of 25.32%. Polar also had an overestimation of TST, with MAE of 45.08 min and MAPE of 13.80%. ActiGraph GT9X results were inconspicuous. The trackers significantly underestimated awake times (WASO + SOL) with weak correlation, r = 0.11–0.57. The highest MAE was 50.35 min and the highest MAPE was 83.02% for WASO for Garmin and ActiGraph GT9X; Polar had the highest MAE of 21.17 min and the highest MAPE of 141.61% for SOL. Garmin showed significant deviations for sleep stages (*p* < 0.045), while Polar only showed significant deviations for sleep cycle (*p* = 0.000), r < 0.50. Garmin and Polar overestimated light sleep and underestimated deep sleep, Garmin significantly, with MAE up to 64.94 min and MAPE up to 116.50%. Both commercial trackers Garmin and Polar did not detect any daytime sleep at all during the MSLT test. The use of the multisport activity trackers for sleep analysis can only be recommended for general daily use and for research purposes. If precise data on sleep stages and parameters are required, their use is limited. The accuracy of the vital data measurement was adequate. Further studies are needed to evaluate their use for medical purposes, inside and outside of the sleep laboratory. The accelerometer ActiGraph GT9X showed overall suitable accuracy in detecting sleep/wake patterns.

## 1. Introduction

Options for monitoring daily human activity have been continuously developed by using activity trackers to determine almost all vital and movement parameters during the day and at night. These analyses became possible with relatively few and very small sensors integrated into wristwatch-sized components, such as photodiodes, triaxial accelerometers, Global Navigation Satellite System (GNSS) sensors, and temperature and barometric pressure sensors. Activities, sporting movements, heart rate, rest and even stress can be recognized, measured and analyzed [1,2]. This integration and the use of algorithms for calculation and evaluation developed quickly with cloud-based data evaluation systems. The steadily increasing number of commercial wearable devices [3] and the resulting increase in available and relevant measurement data are having a lasting and increasingly rapid impact on accuracy [4]. Unfortunately, the scientific study of activity trackers has some limitations, as the commercial systems do not fully disclose all analysis methods. Most devices are essentially a “black box” in relation to data acquisition and processing using proprietary algorithms and artificial intelligence [5]. Nevertheless, the ability to record and assess rest periods, such as night sleep, has been greatly improved in recent years by wearables and activity trackers [6].

Human sleep as a reversible physiological state is essential for health and performance in everyday life [7]. Its functions are not fully understood, despite extensive studies on its influence on energy homeostasis, immune function, cognitive performance and behavior, and the influence on various clinical pictures [8,9,10,11]. Sleep can be measured in multiple dimensions such as quantity, continuity, timing and quality [12,13,14,15]. Sensors have been used to study sleep for decades since the early 1950s. In laboratory settings, polysomnography (PSG) paired with clinical evaluation has been the gold standard to study sleep as well as to diagnose a variety of sleep disorders [16]. In recent years, industry and academia have invested heavily in the development of smaller, less obtrusive and more portable devices for the continuous monitoring of sleep, such as accelerometers or different multi-sensor devices such as activity trackers with heart rate and movement sensors [17]. The goal is to enable data collection in larger groups over longer periods of time and in a more natural environment outside the lab, with lower costs and more readily available participants. However, challenges remain with respect to data acquisition and long-term data evaluation, which may mislead the estimations of health markers due to possible interruptions and missing periods [16]. Accelerometers and commercial activity trackers for monitoring sleep have received increased scientific attention in recent years. Accelerometers for actigraphy use have been developed to such an extent that the accuracy of measuring sleep epochs was 87–99% in relation to the gold standard PSG, but with a specification of sleep of only 28% and 67% [18,19,20,21]. The actigraphy tends to overestimate TST and SE and underestimate SOL and WASO in sleep disorders [22,23,24]. It should also be noted that actigraphy can generally only be used to determine sleep/wake patterns. The detection and further analysis of sleep stages (light, deep or REM sleep) is not possible [25,26].

The scientific investigation of commercial low-cost activity trackers started to increase exponentially about 10 years ago [27]. Fitbit Ultra and Jawbone UP were among the first wrist-worn fitness trackers to be scientifically studied, with moderate accuracy scores compared to the PSG measurement [28,29,30]. Various wearable sleep trackers are currently on the market, such as bracelets, smartwatches and wrist-worn activity trackers, headbands, rings, sensor clips and others [27]. These devices are easy to use and ready to purchase in the consumer market. Most of the consumer wrist-worn activity trackers rely on a similar mechanism of clinical actigraphy that infers wake and sleep patterns from limb movement [31,32]. The newly launched models also incorporate, besides the triaxial accelerometer, other streams of bio signals such as photoplethysmographic (PPG) sensors to measure heart rate (HR) and heart rate variability (HRV) for further vital data (respiratory rate and SpO_2_) and sleep stage analysis [33]. Normal resting HRV is an indication of cardiovascular and autonomic health as well as general fitness. Greater HRV at night has been linked with better sleep quality and reacts to sleep phases [34,35,36]. HRV is also a biofeedback tool for improved relaxation—when a person is under physical or mental stress, parasympathetic activity decreases and sympathetic activity increases, resulting in HR increase and HRV decrease [37,38]. This systematic fact is the basis for determining the respiratory rate, which can be calculated by algorithms depending on the changes in the autonomic nervous system (ANS) using HRV and HF data [39,40,41]. This phenomenon is called respiratory sinus arrhythmia (RSA) [42]. Thus, the respiratory rate is another building block for the assessment of sleep and sleep quality [43]. More recently, it is possible to visualize a sleep hypnogram of a whole night via the evaluation app of the activity tracker manufacturer and to analyze the aggregated sleep parameters such as the total sleep time (TST), wake times and the ratio of the individual sleep stages [44]. The latest validity studies of wrist-worn activity trackers showed clear improvements in the accuracy of the sleep analyses compared to the first studies from 2015 [29,30,44]. However, differences in the degree of accuracy can be seen depending on the brand, the sensors and algorithms used [2,45,46], with accuracies to the gold standard between moderate and good in measuring sleep phases such as TST, WASO, SOL and SE [47].

The analysis of sleep in stages of NREM (light, NREM stages 1 and 2; deep sleep, NREM stage 3) as well as REM sleep is rather underrepresented in the current study situation. Most of the studies show problems with the fragmentation and discrimination of the individual sleep stages during sleep. Liang and Chapa Martell [12] and Liang and Martell [31] showed difficulties of the trackers in the transition phase between the stages of light to REM and deep to awake with a bias of 0–60%, as well as significant deviations in the total time of the measured stages REM and light sleep. It has been found that trackers for sleep patients with sleep disorders are not yet sufficiently developed. Another study showed that the accuracy of determining deep sleep with the tracker was limited, with a medium accuracy of 0.49 [47]. Moreno-Pino et al. [48] found that the tracker recorded significant differences in all sleep stages except in REM. In most studies, light sleep was overestimated and deep sleep was underestimated [12,31,47,48]. Results in the literature show a variable accuracy (moderate to good) of the wrist-worn activity trackers. Most of the research has been carried out on healthy young subjects [6,48,49]. A study of middle-aged to older sleep patients, patients with an average BMI in the obesity range, and patients with sleep disorders or sleep apnea and the isolated usage of nocturnal breathing aids, such as Continuous Positive Airway Pressure (CPAP) devices, can provide an extended view of the accuracy under these conditions.

Only a few sleep validation studies have been conducted with the latest Garmin Forerunner or Fenix models and Polar models with moderate to high accuracy in measuring sleep stages and sleep parameters, with r = 0.60–0.88 for Garmin [2,45,46,50] and r = 0.54–0.90 for Polar [51,52,53,54]. The accelerometer ActiGraph GT9X has been validated several times in the recent past for sleep wake/pattern measurement against PSG with high accuracy of r = 0.85–0.89 [55,56,57,58,59,60], and it is listed as a Food and Drug Administration (FDA)-approved device for clinical use with an AASM recommendation for clinical use in sleep disorders and circadian rhythm sleep–wake disorders [55,56,57,58,59,60].

The aim of this study was to validate sleep stages and parameters as well as vital data measurements of two multisport activity trackers (Garmin Forerunner 945 and Polar Ignite) and one accelerometer (ActiGraph GT9X) against PSG in a laboratory setting. Forty-nine adult sleep patients (30 males/19 females) completed a standardized one-night sleep examination followed by a multiple sleep latency test (MSLT) the following day.

## 2. Materials and Methods

### 2.1. Participants

Forty-nine (30 males/19 females) adult sleep laboratory patients volunteered to participate in the study at the sleep laboratory, Somnolab Dortmund (Dortmund, Germany), between 23 April 2020 and 10 December 2020. The participants were consecutively recruited based on the examination combination of one night’s sleep followed by the MSLT the following day. Descriptive characteristics as well as additional information about the skin and activity scales are shown in Table 1. Twelve participants (9 males/3 females) wore a CPAP during their nocturnal sleep examination. The study population was entirely Caucasian. All participants provided written informed consent to participate in this study.

The study was approved by the Medical Association of Westphalia-Lippe, which is connected to the Westfälische-Wilhelms-University Münster, Germany (file number 2019-611-f-S). Furthermore, the Ethics Committee of the German University for Health and Sport (DHGS) checked and approved the study design (reference number 08/2019.1). In addition, the study was included as a medical trial in the World Health Organization (WHO) Primary Register via the German Clinical Trials Register (DRKS) with the reference number DRKS00021701 and conducted according to the guidelines of the Declaration of Helsinki.

### 2.2. Design and Laboratory Procedures

The study was designed as a laboratory study with various patient complaints relating to their sleep. All participants received a standardized polysomnography to measure sleep over one night and a standardized test assessing daytime sleep propensity, the MSLT, the following day [65,66,67].

On the recording night, participants reported to the laboratory around 2.5 to 3.0 h prior to their typical lights-off time. They underwent in-processing with some vital data measurements such as reaction times, weight, blood pressure and HR, later followed by the application of the PSG electrodes and sensors by the sleep technician. Both multisport activity trackers and the accelerometer were randomly fitted tightly, alternately on the left and right forearm behind the processus styloideus ulnae, according to each device company’s guidelines [68,69]. The beginning and the end of the PSG and activity tracker device data collection periods coincided with the lights-off time and the end of the MSLT. To ensure time synchronization, all devices were connected to the computer or a cellphone app once a day during data upload, using the Network Time Protocol (NTP), which continuously synchronizes to the atomic time of the Physikalisch-Technische Bundesanstalt (Braunschweig, Germany). All recordings were performed in sound-attenuated and temperature-controlled sleeping rooms.

### 2.3. Devices and Sleep Parameters

Laboratory PSG assessments were performed using Embla Somnologica—PSG unit (Embla Somnologica System, Amsterdam, Netherlands; Resmed Inc., Martinsried, Germany), with data sampled and stored at 256 Hz. Electroencephalographic (EEG) (including F3-M2, F4-M1, C3-M2, C4-M1, O1-M2, O2-M1), electrooculographic (EOG), electromyographic (chin EMG) and two-lead electrocardiographic (ECG) measurements were performed. PSG sleep stages were scored in 30 s epochs by an experienced medical technician according to the standards of the American Academy of Sleep Medicine (AASM) rules [70]. Further vital measurements of oxygen saturation (SpO_2_), respiration (respiratory rate) and leg movements were collected to recognize potential sleep disorders according to the guidelines of the AASM [70]. The following PSG measurements were calculated: time in bed (TIB, minutes), total sleep time (TST, minutes), sleep efficiency TST/TIB*100 (SE, %), sleep onset latency (SOL, minutes), wake after sleep onset (WASO, minutes) and time spent in N1, N2, N3 and REM sleep (all in min) according to the AASM [70]. The definitions of the sleep parameters, sleep stages and the vital parameter measurements are summarized in Table 2.

#### 2.3.1. Accelerometer ActiGraph GT9X

The accelerometer ActiGraph GT9X (ActiGraph, Pensacola, FL, USA) (serial number TAS1F28170667) is a wrist-worn triaxial accelerometer on a research-grade level. Depending on its version, it can also be worn on the waist or ankle. Accelerometers are able to detect movement by acceleration of the human body using internal sensors such as an inertial measurement unit (IMU) and wear time sensors [68,69]. The measured raw data were downloaded in the lab using ActiLife software (version 6.13.4, ActiGraph, Pensacola, FL, USA). ActiLife software uses the Cole–Kripke sleep algorithm to calculate sleep parameters. In and out of bed times, TIB, TST, WASO, SOL, SE, Awake time and number of awakenings were calculated [68,69]. Due to the lack of appropriate internal sensors, HR, HRV, SpO_2_ oxygen saturation and respiratory rate could not be determined.

#### 2.3.2. Garmin Forerunner 945

The multisport activity tracker Garmin Forerunner 945 (Garmin Ltd., 2022; Olathe, KS, USA) (ID 3996687672. firmware 5.50, e6bbb98) is able to detect activity and sleep as well as vital data with several sensors, including a triaxial accelerometer, a Global Navigation Satellite System (GNSS) sensor for GPS or GLONASS and a photodiode sensor for photoplethysmographical measurements of HR, HRV, SpO_2_ and respiratory rate. The Garmin device allows tracking of sleep stages (light, deep and REM sleep time) in addition to the sleep parameters (TIB, TST, Awake, SOL and WASO) [71]. The measured raw data were transmitted to the Garmin Connect cloud via Bluetooth and the Internet and analyzed by Garmin using a proprietary sleep assessment algorithm. The scientific investigator could not influence this process and was blind to it. The sleep stages and sleep parameters as well as a hypnogram were displayed in the Garmin Connect app and stored in the Garmin data cloud. In addition, SpO_2_ and respiratory rate data were provided. Respiratory rate can be calculated depending on the changes in the autonomic nervous system (ANS) using HRV and HF data [39,40,41]. Unfortunately, HRV and beat to beat data were not directly provided in the spreadsheet report by the Garmin app [71].

#### 2.3.3. Polar Ignite

The Polar Ignite, the second multisport activity tracker (Polar Electro Oy, 2022; Kempele, Finland) (ID 5D935A29, firmware 2.0.25), uses similar sensors to the Garmin device. The tracker is also able to detect sleep stages such as awake time and light, deep and REM sleep time, as well as the normal sleep/wake patterns TIB, TST, Awake, SOL and WASO. For further detailed sleep analysis, the raw data were automatically transferred to the Polar data cloud. Polar uses the “Nightly Recharge System” as a proprietary algorithm. All analyzed data were displayed in the Polar Flow app. In addition to the hypnogram, three further diagrams were displayed, HRV (RMSSD), beat to beat measurement and the respiratory rate data. The respiratory rate was calculated according to the same principle as the Garmin device, explained in Section 1. The measurement detection was restricted to the first four consecutive hours of sleep detection during sleep. In addition, no SpO_2_ data were calculated or reported in the spreadsheet report of the Polar Flow app [72].

#### 2.3.4. Data Extraction and Scoring

After the MSLT measurement, both multisport activity trackers Garmin and Polar were synchronized with the respective companies’ data cloud app via computer and Internet. After the calculation of sleep parameters, sleep stages and vital data by proprietary algorithms, these data were available as a report for further scientific analysis via the apps Garmin Connect (Garmin Ltd., Olathe, KS, USA, version: 4.37.2.0) and Polar Flow (Polar Electro Oy, Kempele, Finland, version 4.8.0). Garmin and Polar did not provide any raw data due to their company restrictions. It was not possible to extract data directly from the activity trackers. The accelerometer ActiGraph GT9X data were uploaded to the local lab computer software ActiLife with the respective sleep parameter calculation function. ActiGraph GT9X and PSG laboratory software (Somnologica version 3.1), in combination with scoring of an experienced sleep laboratory technician, provided a detailed sleep-related written data report, standardized in accordance with AASM guidelines [72]. All data were extracted from each spreadsheet report and summarized in MS Excel sheets (Microsoft, Redmond, WA, USA, version 2016) for further statistical analysis.

### 2.4. Statistical Analysis

All statistical analyses were performed using MS Excel 2016 and IBM SPSS (version 26.0 Armonk, NY, USA). The significance level was set at *p* < 0.05. Descriptive data analyses of each subject’s physical data were performed, and the normal distribution of all data was assessed using the Kolmogorov–Smirnov test. After all *t*-test analyses, false discovery rate (FDR) corrections were performed to counteract the alpha error summation by multiple testing [73]. The following statistical tests were based on the recommendations for wearable and actigraphy monitoring, validation and assessment selected by [74,75,76,77,78,79]. They were also used in other previous wearable validation studies [28,80,81].

Correlations of sleep parameters and vital data measurements between the gold standard PSG and Garmin, Polar and the accelerometer ActiGraph GT9X results were calculated (two-way random, absolute agreement). Agreement was considered high >0.79, moderate 0.40–0.79 or low <0.40. Difference analyses for sleep parameters and vital data measurements were calculated using the mean absolute error (MAE) and mean absolute percentage error (MAPE) (|[mean difference activity tracker − criterion measurement] × mean criterion measurement ^−1^| × 100). The total deviation from the PSG measurement was calculated and expressed in a boxplot diagram. T-tests followed by FDR correction, one-way ANOVA followed by the Scheffé post hoc test, and effect size calculations in accordance with Cohen [82] were calculated. The interpretation of the effect size was based on Cohen’s d classification: there is a small effect from a value of d = 0.20, a medium effect from d = 0.50 and a large effect from d = 0.80, and Eta^2^ (ή^2^) with >0.01 is a small effect, >0.06 a medium effect and >0.14 a large effect [82]. The level of agreement (LoA) was calculated for sleep parameter measurements between the wearable devices’ results and PSG measurements. LoA was assessed as described by Bland–Altman and was expressed using a Bland–Altman diagram [83].

Statistical power (1-ß error probability) was determined post hoc using G*Power (University Düsseldorf, Düsseldorf, Germany) for correlation, *t*-test and ANOVA analysis [84,85].

## 3. Results

### 3.1. Vital Data Measurement

HR measurements (HR, HRV and beat to beat) showed strong correlations with r > 0.92 (*p* = 0.000) in general between each multisport activity tracker and the PSG-derived two-lead ECG. The difference analysis of all HR measurements showed a small MAE <12.67 beats per minute/MAPE <1.93%. HRV and beat-to-beat measurements could only be calculated for Polar Ignite; Garmin did not present these data even though it is used to calculate sleep data. The Garmin oxygen saturation measurement showed low to moderate correlations with r < 0.52; the SpO_2_ minimum analysis was particularly noticeable with r = 0.27 (*p* = 0.057) and MAE of 4.58/MAPE of 5.38%. Statistical power for correlation analysis was determined post hoc with 47–99%. The respiratory rate measurements of both devices were unobtrusive. Paired *t*-test analysis showed for all vital data measurements no significant differences (*p* = 0.051–0.992). Further boxplot analysis of all vital data confirmed the calculated results.

### 3.2. Sleep Parameters

The measurement of the sleep parameters showed different results than the vital data analysis. Overall, the calculated correlations were low to moderate with r = 0.11–0.63 (*p* = 0.000–0.569). The TST and the awake phases (Awake, WASO and SOL) were particularly noticeable. The accelerometer calculation showed the highest values with r > 0.52 (*p* < 0.009). The difference analysis of all sleep parameters showed, especially for the awake phases for both activity trackers and the TST for Garmin, high values of MAE up to 84.63 min/MAPE up to 141.61%. The accelerometer again showed lower values for all measurements (Table 3). 

The calculated ANOVA values showed a similar picture. Garmin and Polar showed significant differences in all awake phases (*p* = 0.000) with medium to large effect up to ή^2^ = 0.429 except SOL on the Polar watch. Garmin also showed significant differences in TST (*p* = 0.000) with large effect ή^2^ = 0.281. ActiGraph GT9X was unremarkable in all calculations, with the exception of SOL (*p* = 0.000) with medium effect ή^2^ = 0.131. The differences between the multisport activity trackers in these measurements were also significant for TIB, TST, Awake and SOL (*p* = 0.000) with medium to large effect (Table 4). Statistical power for ANOVA calculations was determined post hoc with 49–100%.

**Figure 1:**
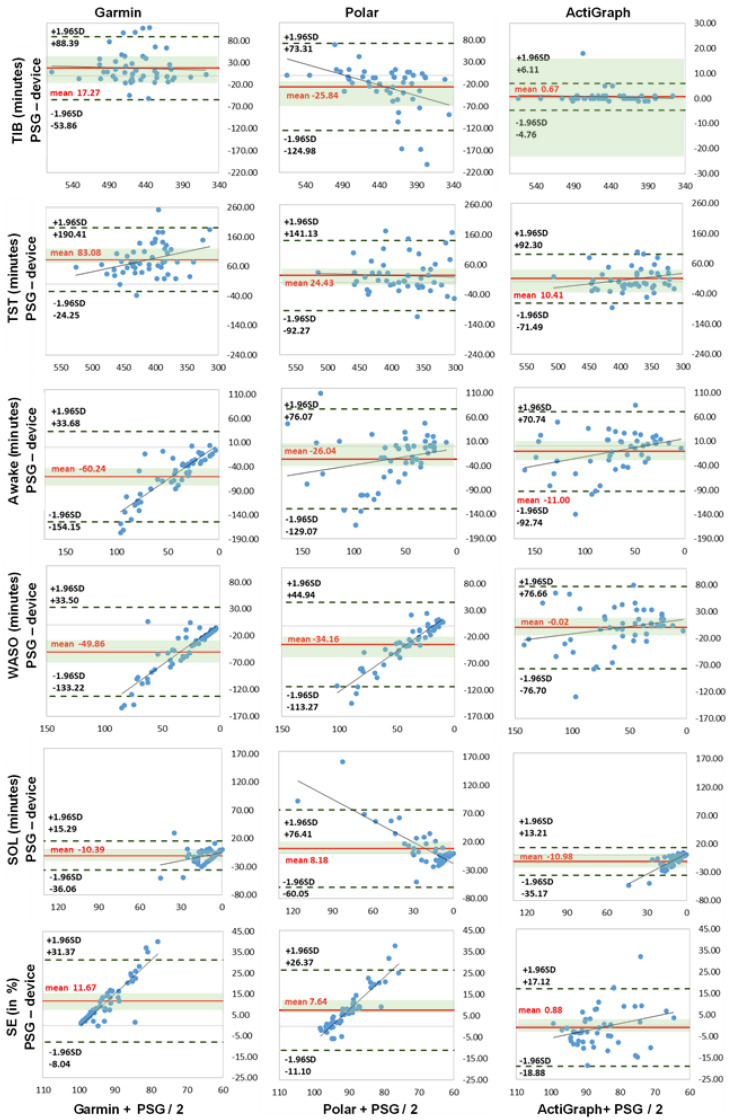
Bland–Altman plots of sleep parameters (*n* = 49). TIB = time in bed, TST = total sleep time, Awake = awake time (WASO + SOL), WASO = wake after sleep onset, SOL = sleep onset latency (all expressed in minutes), SE = sleep efficiency (in %). *x*-axis represents the mean values of the device and PSG; *y*-axis represents the differences between the PSG and the device; dashed black line represents the upper and lower limit of agreement (mean +/− 1.96 SD); solid red line represents the mean value of difference; solid blue line represents the trend; shaded green area represents 95% CI (confidence interval) of mean difference.

**Figure 2:**
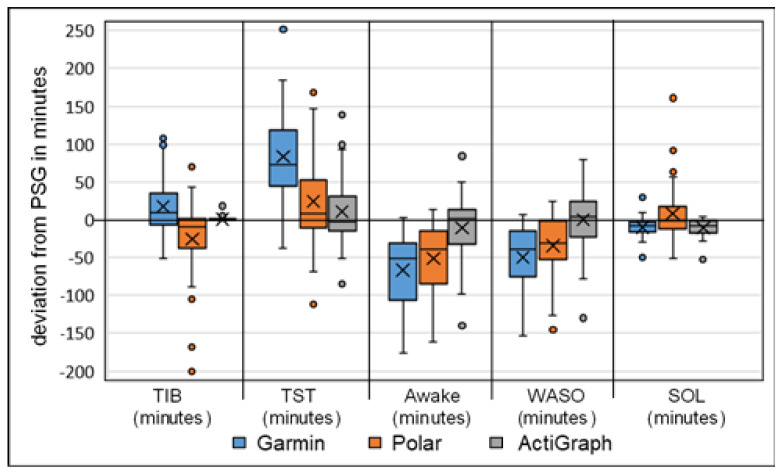
Boxplot analysis of calculated deviation in sleep parameters, Garmin, Polar and ActiGraph GT9X against the gold standard PSG (*n* = 49), TIB = time in bed, TST = total sleep time, Awake = awake time (WASO + SOL), WASO = wake after sleep onset, SOL = sleep onset latency (all expressed in minutes); the x represents the mean value of deviation.

The Bland–Altman and boxplot diagrams show the greater fluctuation range of activity trackers Garmin and Polar for TST, awake parameters and SE (Figure 1 and Figure 2). In particular, the LoA differences between the diagrams showed deviations up to 190 min for Garmin and Polar. The accelerometer analysis was comparatively lower for all measurements. This supports the previous calculations.

### 3.3. Sleep Stages

The analysis of the sleep stages yielded logical results based on the sleep measurement analysis. Due to device and sensor limitations, the accelerometer ActiGraph GT9X did not calculate any sleep stage. Overall, the differences in sleep stages between the two activity trackers and the PSG are slightly lower than in the sleep parameter analysis. With the exception of deep sleep, higher values were calculated here, with MAE up to 47.33 min/MAPE up to 116.50% for both (Table 5).

The correlation calculation shows similar results, especially with the deep sleep values of r = 0.11–037 (*p* = 0.008–0.432). With the paired *t*-test analysis, Garmin showed for all stages significant values (*p* < 0.045) with medium to large effect sizes up to d = 1.840, except for REM sleep, against the PSG. Polar showed significant results in sleep cycle only (*p* = 0.000) with large effect size, d = 1.036 (Table 6). Statistical power for *t*-test calculations was determined post hoc with 42–100%.

**Figure 3:**
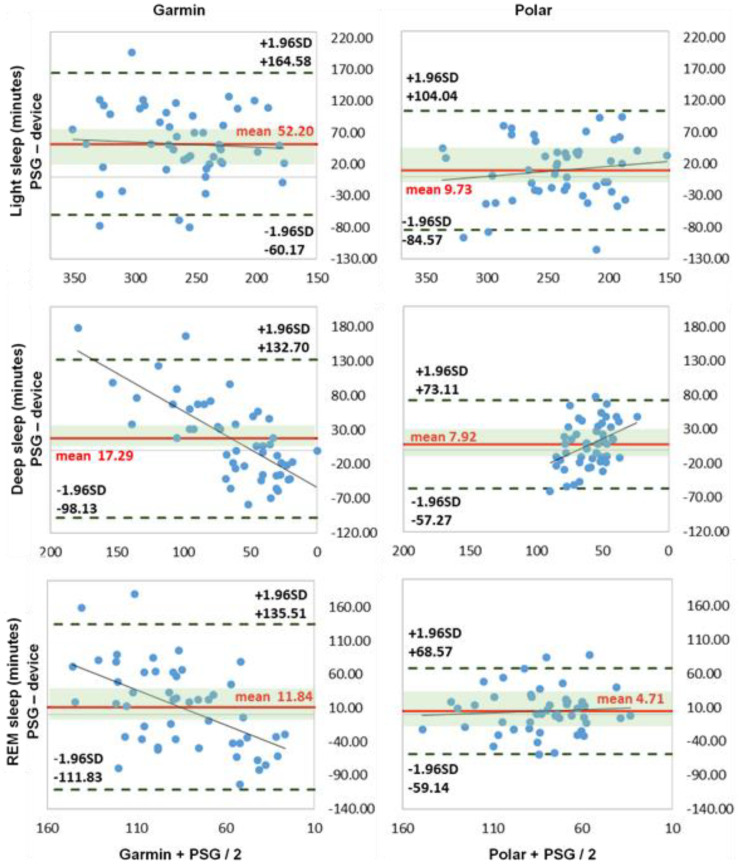
Bland–Altman plots of sleep stages (*n* = 49). NREM, light sleep (NREM1 + NREM2), deep sleep (SWS, NREM3) and REM sleep = rapid eye movement sleep (all expressed in minutes). *x*-axis represents the mean values of the device and PSG; *y*-axis represents the differences between the PSG and the device; dashed black line represents the upper and lower limit of agreement (mean +/− 1.96 SD); solid red line represents the mean value of difference; solid blue line represents the trend line; shaded green area represents 95% CI (confidence interval) of mean difference.

**Figure 4:**
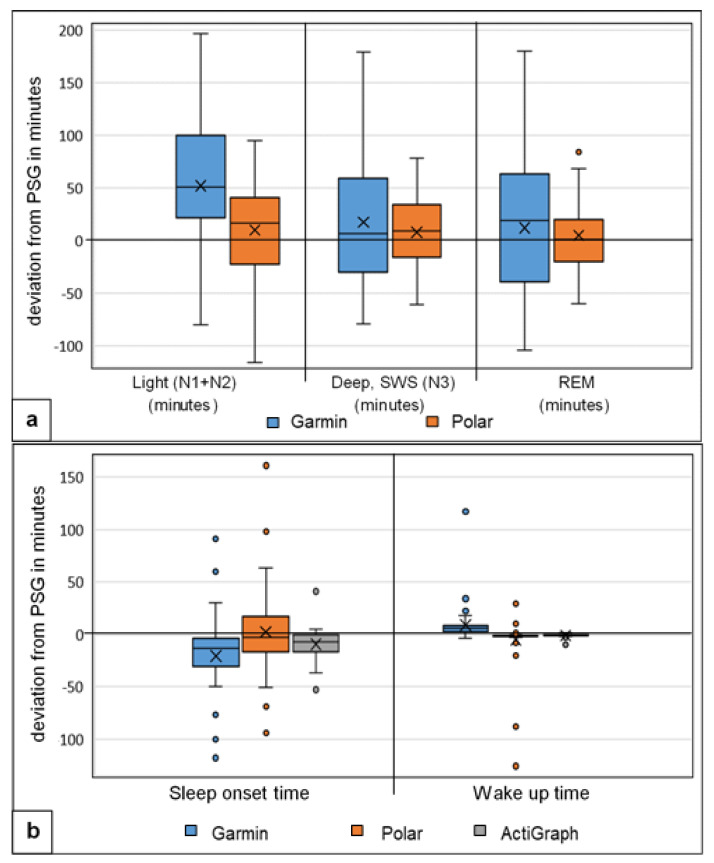
Boxplot analysis of calculated deviation (**a**) in sleep stages, Garmin and Polar against the gold standard PSG (*n* = 49), Light = light sleep, Deep = slow-wave sleep (SWS), REM = rapid eye movement sleep (all expressed in minutes) and (**b**) in sleep onset time = start of sleep and wake-up time = end of sleep (deviation expressed in minutes), Garmin, Polar and accelerometer (ActiGraph GT9X) against the gold standard PSG (*n* = 49); the x represents the mean value of deviation.

The Bland–Altman diagrams and the boxplot analysis show differences between the two activity trackers Garmin and Polar, as well as the larger differences measured in light and deep sleep with Garmin (Figure 3 and Figure 4a,b).

### 3.4. Hypnogram Analysis

**Figure 5:**
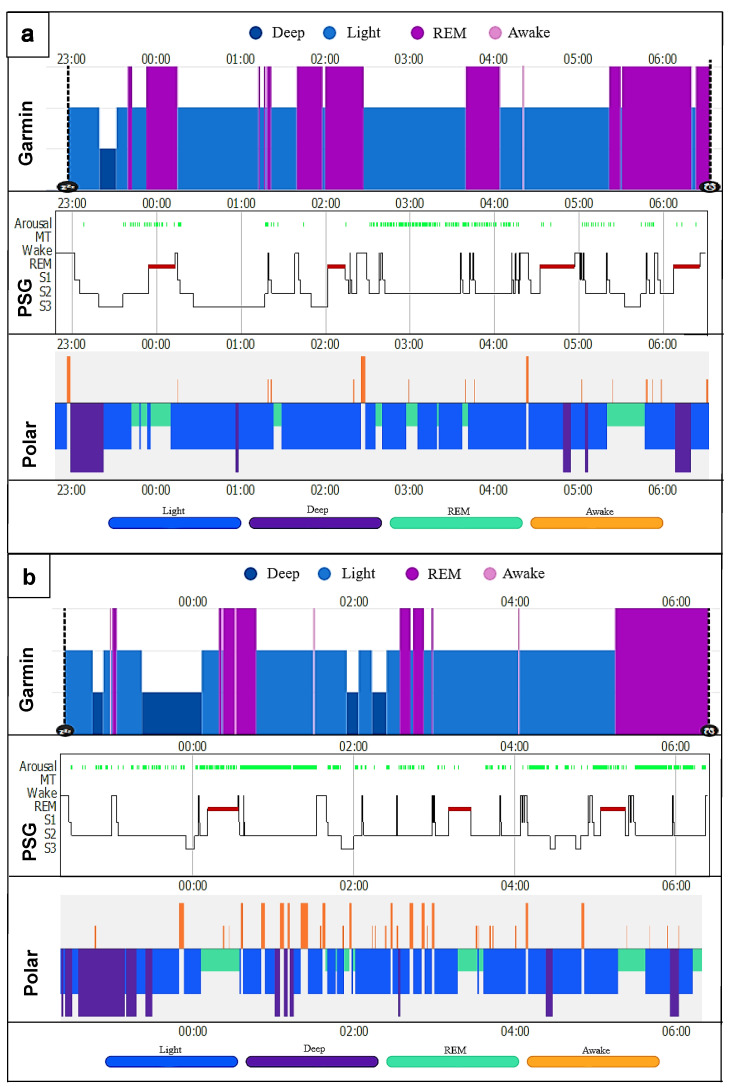
Night sleep hypnograms (Garmin, PSG, Polar); (**a**) CPAP patient (male); (**b**) sleep patient (female). Arousal = partial, temporary or complete wake-up reaction with sleep-disrupting effect [66]; MT = movement time; Wake = awake time; REM = rapid eye movement sleep; S1–S3 represent NREM1–NREM3 (N1 + 2 = light sleep; N3 = deep sleep [70]). *x*-axis represents the time in hours; *y*-axis represents the respective sleep stage.

Both commercial activity trackers have problems recognizing the exact distribution in time of the sleep stages. Two hypnogram triads (Garmin, PSG and Polar) were put together as an example (Figure 5a,b). The diagrams show that neither could adequately represent the transitions from light to deep sleep or light sleep to REM sleep and back to the awake state. The exact lengths and actual times of the start and end of the individual stages were mostly not reproduced correctly. Garmin had bigger problems than Polar, especially in detecting and distinguishing between light sleep and awake, as well as REM sleep and light sleep. Both had significant problems recognizing deep sleep. Comparing the rest of the hypnograms, similar results were seen, regardless of the use of CPAP. However, the more restless the patient’s sleep with regard to movement in bed, the number of arousals and the frequent change from awake to light sleep, the more mismatches were recognizable (Figure 5b).

### 3.5. MSLT Measurement

Unfortunately, the statistical evaluation of the MSLT measurement was not possible because the activity trackers Garmin and Polar did not calculate any data during this time period. Only a visual, schematic, imprecise analysis of the Garmin hypnogram would be possible, since Garmin provides additional visual data via the manual adjustment of the wake-up time function. With Polar, this was not yet possible at the time of the study; currently, it is possible due to further software updates. Due to the lack of data from the multisport activity trackers, it was decided not to further investigate the existing ActiGraph data. The main result is therefore that the multisport activity trackers Garmin and Polar currently do not indicate daytime sleep, although the sensors for it are available.

## 4. Discussion

The aim of this study was to assess and validate the accuracy of two multisport activity trackers (Garmin and Polar) and one accelerometer (ActiGraph GT9X) in measuring sleep stages, sleep-related measurements and vital parameters of sleep patients in a laboratory setting. As the main findings, the study showed accurate measurement of the vital data, such as HR, HRV, respiratory rate and SpO_2_, with the exception of the SpO_2_ minimum measurement by the Garmin device. In contrast, the sleep-related measurements were far less accurate for the two multisport activity trackers Garmin and Polar. Both trackers tending to overestimate TST and underestimate awake (SOL and WASO). In the further analysis of the sleep stages, this resulted in larger deviations of both trackers in light and deep sleep, mainly in the overestimation of light sleep and underestimation of deep sleep, less in REM sleep. Garmin presented a larger and more significant deviation than Polar. The accuracy of the accelerometer to detect sleep/wake patterns was adequate with the only restrictions of the significant deviation and underestimation in SOL.

Sleep stages differ from each other in physiological terms, such as breathing, autonomic nervous system (ANS) reaction and body movement. These behavioral differences and their physiological responses are driven by a coupling between central nervous system activity and ANS activity that can provide the theoretical framework for sleep calculations without EEG-based systems [86]. The examination of the vital data measurement was the logical prerequisite for further validation of the sleep data calculations. Unfortunately, only HRV data were provided by Polar and SpO_2_ data by Garmin. The measured vital data HR, HRV and the respiratory rate showed high agreement with the PSG for both activity trackers, r > 0.92. The differentiation calculation analysis showed similar results with MAE of 5.82 and MAPE up to 8.72% of Polar HRV, but with a small mean of −1.93 min. The boxplot analysis supports these results and shows the greatest variance in HRV and beat to beat, which could have a negative impact on the calculation of the sleep-related measurements and the calculation of the sequence algorithm in the sleep stages. The less accurate agreement r = 0.27 (*p* = 0.057) of the SpO_2_ minimum measurement of the Garmin device has no direct influence on the sleep stage calculation. Recent studies have shown mostly similar results with a high level of agreement between the measured vital data (HR, HRV and respiratory rate) against the PSG and ECG measurements [1,37,87,88,89].

The TIB and TST measurements showed medium to high correlation values with r > 0.42 (*p* > 0.003). TIB results showed no significant differences, with the highest values of MAE 34.00 min and MAPE of 7.53% for Polar. Garmin generally overestimated the time by 17.27 min, and Polar underestimated it by −25.84 min. In the TST measurement, the differences between the Garmin device and the PSG were significantly noticeable (*p* = 0.000; d = 0.3). The more detailed difference calculation yielded MAE 84.63 min with MAPE of 25.32% for Garmin, MAE 45.08 min and MAPE of 13.80% for Polar, and MAE of 31.39 min and MAPE of 9.33% for the accelerometer (ActiGraph GT9X). The Bland–Altman and boxplot analysis showed clear overestimations by Garmin with 83.03 min and Polar with 24.03 min, and the accelerometer (ActiGraph GT9X) was negligible. This is in line with the findings of studies with an overestimation of up to 37 min for TST [2,22,23,47,90,91,92].

For the Awake measurements, all devices showed weaker correlation values r = 0.11–0.57 (*p* = 0.000–0.098). Garmin and the accelerometer (ActiGraph GT9X) showed the highest deviations for the WASO with MAE of 50.35 min and MAPE of up to 83.02%. Polar showed the highest deviations for SOL with MAE of 21.71 min and MAPE of up to 141.61%. Garmin had the highest differences and the accelerometer (ActiGraph GT9X) had differences in SOL only. All results were significantly different (*p* < 0.010) and with medium to large effect sizes of up to ή^2^ = 0.429, except the accelerometer (ActiGraph GT9X) for WASO and Polar for SOL. All trackers mostly underestimated the awake times. The large effect size underlines the underestimation of the mentioned awake phases and supports the MAE/MAPE difference analysis. The results for the activity trackers are in agreement with the current study situation with significant underestimation of WASO and SOL [2,20,22,58,90,91,92,93]. The current accelerometer studies differ only slightly in the SOL measurements; the deviations were significantly less than in this study [22,23]. This led to longer TST times with the two multisport activity trackers, since the TIB did not show any major deviations. Furthermore, this affected sleep efficiency, since less awake time leads to higher SE results. However, in this study, the differences were mostly significant and the mean deviations were significantly larger, especially in awake time measurements [91,92].

The sleep phase calculation showed significant differences for the Garmin measurement (*p* < 0.045) with medium to large effect sizes, d = 0.658–1.840. Polar was not significantly different here. The correlation values for all sleep stages (light, deep, REM sleep) and the sleep cycles were low to moderate (r = 0.02–0.50) for both activity trackers. Garmin had the highest deviation in all three sleep stages, with MAE up to 64.94 min and MAPE up to 116.50%; Polar showed smaller values, with MAE up to 40.14 min and MAPE up to 87% for light and deep sleep. Light sleep was overestimated and deep sleep underestimated by both trackers. The large effect size results for Garmin underline these significant difference results. The results are in line with existing studies by [12,27,33]. Specifically, [48] showed congruent results, with significant differences and overestimation of light sleep and underestimation of deep sleep. Garmin had problems in acquiring and calculating the exact sleep stages with significant deviations. The deviations from Polar, on the other hand, were not quite as large. The overestimation of light sleep is directly related to Garmin’s measured earlier time of falling asleep in combination with the underestimation of awake times. A possible pre-rest phase could have been rated as sleep and thus led to higher TST and light sleep times. Polar showed a slightly larger error range for all stages, which, however, did not lead to tendencies in one direction due to the distribution. The relatively small mean deviations from the PSG can therefore easily be explained by the arithmetic mean.

Neither multisport activity trackers recorded the multiple sleep latency test (MSLT) on the following day after night sleep measurement. Neither tracker recognized daytime sleep times after rise time from bed. The night sleep time window had to be set for both trackers (Garmin and Polar) before use, usually between 10:00 p.m. and 08:00 a.m. Sleep times on the following day past 08:00 a.m. or after wake-up time were not included in the sleep calculation by the tracker. During the study, it was possible to adjust the sleep onset and wake-up time after the night sleep with the Garmin device only. This lengthened the hypnogram and added to the night sleep calculation. There was no defined calculation, delineation or interpretation of sleep stages for the MSLT test at all. No distinction was made in the calculation between night and day sleep, so it was not useable. Polar did not provide any data at all. The accelerometer (ActiGraph GT9X) recorded MSLT data, which were not further statistically evaluated. So far, no current studies on MSLT testing with commercial activity trackers are known.

Possible influence on measurement or calculation inaccuracies can arise from the composition of the sample. Mostly older normal sleep patients (55.01 +/− 10.19 years) with and without sleep disorders and sleep apnea (including twelve CPAP patients) were chosen. The high BMI (30.57 +/− 6.32 kg*m^−2^) was particularly noticeable. The quantity and quality of sleep can change profoundly across the lifespan [49,94]. Older people commonly have difficulty falling asleep and staying asleep. The sleep architecture and depth may change with ageing. Behavioral changes and daytime napping can impact nocturnal sleep. Older people spend more time in stages N1 and N2 (light sleep). This leads to multiple awakenings during the night, which is described as sleep fragmentation with ageing. With increasing age, the percentage of REM sleep decreases in elderly women; in contrast, the slow-wave sleep significantly decreases in elderly men [95,96,97]. The fragmentation of sleep in the elderly and the longer periods of wakefulness may affect the calculation, since studies involving healthy young subjects had much smaller deviations [33,47] than this study. The assumption is that the sleep algorithm has possible problems with the calculation. It should also be noted that self-learning functions within the sleep algorithms could achieve better measurement results in the long term for vital data such as HR and HRV and also for sleep parameters and sleep stages. Because of the usable number of devices during the study, only two trackers were used once per sleep patient. If each participant uses their own device and wears it for a longer time, a self-learning effect could occur and could lead to measurement improvements [16,98,99].

As a limitation of this study, only two multisport activity trackers and one accelerometer were included. Therefore, a general statement about all wearable activity trackers and accelerometers for actigraphy use is not intended. The sleep data used were extracted directly from the spreadsheet reports of the manufacturers’ apps (Garmin Connect and Polar Flow). Even when asked, Garmin and Polar were not able to provide the raw data. Therefore, it was not known exactly which formulas and algorithms or artificial intelligence were used in the calculation. Furthermore, the epoch-by-epoch analysis of the sleep measurements could possibly vary, since changes in the manufacturers’ cloud-based calculations are not communicated directly to the user. It should be noted that only one night’s sleep with MSLT was conducted in a laboratory setting. With multiple-night studies including also non-laboratory settings, a larger dataset could contribute to an even more accurate validation analysis. Finally, an ad hoc sample was analyzed in this study. Therefore, an a priori sample size estimation via G*Power was not possible. Therefore, the results must be considered with a certain degree of caution. Still, based on the post hoc G*Power analysis, the sample size is sufficient to allow a solid (Power [1-β err prob] 42–100%) evaluation; additionally, comparable studies have used a similar number of subjects (*n* = 26–56) [22,47,50,56,90].

## 5. Conclusions

In summary, this study showed high accuracy in measurements of vital data (HR, HRV, SpO_2_ and respiratory rate) during one night of sleep examination. When measuring the sleep-relevant parameters (TIB, TST, Awake (SOL and WASO) and SE), the multisport activity trackers showed significant differences from the PSG measurement with medium to large effect sizes, with the exception of the TIB measurement. TST was overestimated and awake times underestimated; Garmin deviated significantly more than Polar. The accelerometer ActiGraph GT9X showed significant deviations in the SOL measurement only with medium effect size. In the sleep stage measurement (light, deep, REM sleep), the detection and measurement of time spent in individual sleep stages and states were particularly problematic for both commercial trackers. Again, Garmin deviated more than Polar, with significant differences and medium to large effect sizes by underestimating deep sleep und overestimating light sleep. Neither multisport activity tracker detected or calculated the multiple sleep latency test (MSLT). The accelerometer ActiGraph GT9X confirmed the known accuracies to determine sleep/wake patterns and could be used as a possible reference for further scientific studies under free-living conditions.

The use of the multisport activity trackers (Garmin Forerunner 945 and Polar Ignite) for sleep analysis can only be recommended for everyday use and general purposes to give important feedback to active populations and for research purposes where large sample sizes are needed. If precise data on sleep stages and parameters are required, their use is limited. Further studies are needed to evaluate their use for medical purposes, both inside and outside of the sleep laboratory. Follow-up studies are recommended with the newest devices, including elderly sleep patients with sleep disorders and other physical limitations such as obesity BMI status.

## Figures and Tables

**Table 1:** Descriptive characteristics of participants (*n* = 49).

Descriptive Characteristic		Male (*n* = 30)	Female (*n* = 19)	Total (*n* = 49)
Age (year)	Mean +/− SD	52.10 +/− 9.63	59.58 +/− 9.56	55.01 +/− 10.19
	Range	27–66	37–74	27–74
Height (meter)	Mean +/− SD	182.7 +/− 6.38	165.8 +/− 5.50	176.2 +/− 10.24
	Range	171–198	158–178	158–198
Weight (Kg)	Mean +/− SD	98.10 +/− 13.77	89.37 +/− 25.40	94.71 +/− 19.36
	Range	75.20–125.00	60.60–150.40	60.60–150.40
BMI (kg × m^−2^)	Mean +/− SD	29.43 +/− 4.23	32.37 +/− 8.49	30.57 +/− 6.32
	Range	21.63–41.04	22.61–52.04	21.36–52.04
Underweight (BMI < 18.5)		0	0	0
Normal (BMI 18.5–24.9)		4	4	8
Pre-obesity (BMI 25.0–29,9)		11	5	16
Obesity (BMI > 29.9)		15	10	25
Skin color type	Mean +/− SD	2.60 +/− 0.62	2.37 +/− 0.76	2.51 +/− 0.68
	Range	1.0–4.0	1.0–4.0	1.0–4.0
Activity level	Mean +/− SD	4.9 +/− 2.59	4.63 +/− 2.71	4.80 +/− 2.61
	Range	0.0–9.0	1.0–9.0	0.0–9.0

BMI = Body Mass Index, scale acc. [61], skin color type = Fitzpatrick scale with 6 stages [62], activity level = classification according to Ross and Jackson extended by the NASA scale with 10 stages [63,64].

**Table 2:** Definitions of sleep parameters, sleep stages and vital parameters.

**Sleep Parameters**	**Definition**
TIB	Time in bed in minutes from lights off to lights on.
TST	Total sleep time in minutes of NREM (N1, N2 and N3) + REM from lights off to lights on in the morning.
Awake	Total time in minutes in absence of any NREM stage and REM from lights off to lights on (WASO + SOL).
WASO	Time in minutes of wakefulness after sleep onset.
SOL	Time in minutes from lights off to the first epoch of sleep scored.
SE	Percentage of sleep time from lights off to lights on (TST/TIB × 100).
Sleep cycle	One sleep cycle consists of an individual sequence of NREM and REM sleep, expressed in total numbers.
**Sleep Stages**	**Definition**
Light sleep	Sleep stages NREM1 + NREM2, expressed in minutes.
Deep sleep	Sleep stage NREM3 (also termed slow-wave sleep; SWS), expressed in minutes.
REM sleep	Characterized by rapid eye movements and EMG decrement, expressed in minutes.
**Vital Parameters**	**Definition**
HR	Heart rate, measured in beats per minute.
HRV	Heart rate variability, RMSSD (root mean square of successive differences) measured in 5 min intervals, expressed in milliseconds.
Beat to beat	Average delta of R–R intervals in milliseconds.
SpO_2_	Measurement of arterial oxygen saturation in percent %.
Respiratory rate	Defined as respiratory cycles per minute.

**Table 3:** Sleep parameters deviation calculation of mean absolute error (MAE) and mean absolute percentage error (MAPE) (*n* = 49).

Sleep Parameters		MAE	MAPE %	Min	Max	SD +/−
TIB (in minutes)	Garmin vs. PSG	26.94	6.32	0.00	110.00	29.67
	Polar vs. PSG	34.00	7.53	0.00	201.00	45.39
	ActiGraph GT9X vs. PSG	0.92	0.21	0.00	18.00	2.70
	Garmin vs. Polar	49.22	13.48	0.00	197.00	51.82
TST (in minutes)	Garmin vs. PSG	84.63	25.32	0.00	252.00	52.28
	Polar vs. PSG	45.08	13.80	1.00	172.00	45.60
	ActiGraph GT9X vs. PSG	31.39	9.33	1.00	139.00	29.17
	Garmin vs. Polar	60.29	17.43	2.00	200.00	53.29
Awake (in minutes)	Garmin vs. PSG	60.53	76.03	3.00	176.00	47.55
	Polar vs. PSG	50.04	41.06	14.00	188.00	39.77
	ActiGraph GT9X vs. PSG	31.24	64.89	0.00	140.00	29.43
	Garmin vs. Polar	37.84	70.81	1.00	177.00	37.76
WASO (in minutes)	Garmin vs. PSG	50.35	83.02	5.00	154.00	41.94
	Polar vs. PSG	38.24	62.38	0.00	145.00	36.43
	ActiGraph GT9X vs. PSG	28.51	84.79	0.00	130.00	26.47
	Garmin vs. Polar	18.18	74.83	2.00	44.00	10.38
SOL (in minutes)	Garmin vs. PSG	12.16	72.09	0.00	50.00	11.41
	Polar vs. PSG	21.71	141.61	1.00	161.00	28.40
	ActiGraph GT9X vs. PSG	11.67	70.41	0.00	53.00	11.67
	Garmin vs. Polar	24.02	105.31	0.00	163.00	34.62
SE (in %)	Garmin vs. PSG	11.67	15.30	0.15	40.06	10.05
	Polar vs. PSG	8.68	11.56	0.22	37.81	8.60
	ActiGraph GT9X vs. PSG	6.99	8.65	0.03	32.22	5.94
	Garmin vs. Polar	4.44	4.78	0.12	11.26	2.61

MAE = mean absolute error (respective unit), MAPE = mean absolute percentage error (in %), min = minimum (absolute in respective unit), max = maximum (absolute in respective unit), SD = standard deviation (respective unit), TIB = time in bed, TST = total sleep time, Awake = awake time (WASO + SOL), WASO = wake after sleep onset, SOL = sleep onset latency (all expressed in minutes), SE = sleep efficiency (in %).

**Table 4:** Sleep parameters of Garmin Forerunner 945, Polar Ignite and ActiGraph GT9X and their comparison with the gold standard PSG using one-way ANOVA (*n* = 49).

Sleep Parameters	F-Value (*p*-Value)/ ή^2^-Value	Devices	Mean Diff.	SD+/−	*p*-Value	95% CI	
						Lower	Upper
TIB (in minutes)	F = 6.758 (*p* = 0.000)/ ή^2^ = 0.096	Garmin vs. PSG	17.27	36.29	0.367	−10.04	44.58
		Polar vs. PSG	−25.84	50.58	0.072	−53.15	1.47
		ActiGraph GT9X vs. PSG	0.67	2.77	1.000	−26.64	27.98
		Garmin vs. Polar	43.11	57.12	0.000	15.79	70.41
TST (in minutes)	F = 25.021 (*p* = 0.000)/ ή^2^ = 0.281	Garmin vs. PSG	83	54.76	0.000	53.49	112.67
		Polar vs. PSG	24.43	59.54	0.147	−5.16	54.02
		ActiGraph GT9X vs. PSG	10.41	41.78	0.805	−19.18	40.00
		Garmin vs. Polar	59	55.12	0.000	29.06	88.24
Awake (in minutes)	F = 48.024 (*p* = 0.000)/ ή^2^ = 0.429	Garmin vs. PSG	−60.25	47.92	0.000	−81.73	−38.76
		Polar vs. PSG	−26.04	52.57	0.010	−47.53	−4.55
		ActiGraph GT9X vs. PSG	−11.00	41.70	0.556	−32.49	10.49
		Garmin vs. Polar	−34.20	41.14	0.000	−55.69	−12.72
WASO (in minutes)	F = 36.138 (*p* = 0.000)/ ή^2^ = 0.361	Garmin vs. PSG	−49.86	42.53	0.000	−66.50	−33.22
		Polar vs. PSG	−34.16	40.36	0.000	−50.80	−17.52
		ActiGraph GT9X vs. PSG	−0.02	39.12	1.000	−16.66	16.62
		Garmin vs. Polar	−15.69	13.92	0.073	−32.34	0.95
SOL (in minutes)	F = 9.612 (*p* = 0.000)/ ή^2^ = 0.131	Garmin vs. PSG	−10.39	13.10	0.000	−14.15	−6.63
		Polar vs. PSG	8.12	34.81	0.292	−3.69	19.93
		ActiGraph GT9X vs. PSG	−10.98	12.34	0.000	−14.52	−7.43
		Garmin vs. Polar	−18.51	37.85	0.000	−30.32	−6.70
SE (in %)	F = 39.407 (*p* = 0.000)/ ή^2^ = 0.381	Garmin vs. PSG	14.86	10.05	0.000	10.81	18.91
		Polar vs. PSG	10.83	9.56	0.000	6.78	14.88
		ActiGraph GT9X vs. PSG	2.31	9.19	0.461	−1.74	6.36
		Garmin vs. Polar	4.03	3.21	0.052	−0.02	8.08

TIB = time in bed, TST = total sleep time, Awake = awake time (WASO + SOL), WASO = wake after sleep onset, SOL = sleep onset latency (all expressed in minutes), SE = sleep efficiency (in %), mean Diff. = mean difference, SD = standard deviation, 95% CI = confidence interval with lower and upper limit.

**Table 5:** Sleep stage deviation calculation of mean absolute error (MAE) and mean absolute percentage error (MAPE) (*n* = 49).

Sleep Stages		MAE	MAPE %	Min	Max	SD +/−
Light sleep (in minutes)	Garmin vs. PSG	64.94	30.08	0.00	197.00	42.01
	Polar vs. PSG	40.14	18.24	1.00	116.00	27.69
	Garmin vs. Polar	57.98	26.75	1.00	239.00	49.89
Deep sleep (in minutes)	Garmin vs. PSG	47.33	116.50	0.00	179.00	38.55
	Polar vs. PSG	28.06	87.36	2.00	78.00	19.09
	Garmin vs. Polar	54.04	91.66	1.00	189.00	40.89
REM sleep (in minutes)	Garmin vs. PSG	53.10	97.54	4.00	180.00	35.29
	Polar vs. PSG	24.80	49.13	0.00	88.00	21.37
	Garmin vs. Polar	45.69	61.64	1.00	145.00	33.33
Sleep cycle (total number)	Garmin vs. PSG	1.14	32.66	0.00	4.00	1.00
	Polar vs. PSG	1.12	33.25	0.00	4.00	0.99
	Garmin vs. Polar	1.04	22.41	0.00	3.00	0.93

MAE = mean absolute error (respective unit), MAPE = mean absolute percentage error (in %), min = minimum (absolute in respective unit), max = maximum (absolute in respective unit), SD = standard deviation (respective unit), Light = light sleep, Deep = slow-wave sleep (SWS), REM = rapid eye movement sleep (all expressed in minutes), sleep cycle = total number of sleep cycles.

**Table 6:** Sleep stages of Garmin Forerunner 945, Polar Ignite and ActiGraph GT9X and their comparison with the gold standard PSG (*n* = 49).

Sleep Stages		Mean Diff.	SD+/−	t-Value *z-Value*	*p*-Value	d-Value	95% CI	
							Lower	Upper
Light sleep (in minutes)	Garmin vs. PSG	52.20	57.34	6.374	0.000	1.840	26.91	77.50
	Polar vs. PSG	9.73	48.12	1.416	0.637	0.409	−15.56	35.03
	Garmin vs. Polar	42.47	63.87	4.654	0.000	1.343	17.18	67.76
Deep sleep (in minutes)	Garmin vs. PSG	17.29	58.88	2.055	0.045	0.593	0.37	34.20
	Polar vs. PSG	7.92	33.26	1.667	0.632	0.481	−12.51	28.34
	Garmin vs. Polar	9.37	67.57	0.970	0.337	0.280	−10.04	28.78
REM sleep (in minutes)	Garmin vs. PSG	11.84	63.10	1.313	0.351	0.379	−8.31	31.98
	Polar vs. PSG	4.71	32.58	1.013	0.846	0.292	−15.43	24.86
	Garmin vs. Polar	7.12	56.48	0.883	0.683	0.255	−13.02	27.27
Sleep cycle (total numbers)	Garmin vs. PSG	0.76	1.63	*−2.981z*	0.003	0.658	0.20	1.31
	Polar vs. PSG	1.10	1.37	*−4.572z*	0.000	1.036	0.55	1.65
	Garmin vs. Polar	−0.35	1.36	*−1.797z*	0.072	0.318	−0.90	0.20

Light = light sleep (NREM1 + NREM2), Deep = slow-wave sleep (SWS, NREM3), REM = rapid eye movement sleep (all expressed in minutes), sleep cycle = total number of sleep cycle, z represents the z-value according to Wilcoxon test, mean Diff. = mean difference, SD = standard deviation, 95% CI = confidence interval with lower and upper limit.

## Data Availability

The data are not yet publicly available.
